# Binuclear Cu complex catalysis enabling Li–CO_2_ battery with a high discharge voltage above 3.0 V

**DOI:** 10.1038/s41467-023-36276-8

**Published:** 2023-02-01

**Authors:** Xinyi Sun, Xiaowei Mu, Wei Zheng, Lei Wang, Sixie Yang, Chuanchao Sheng, Hui Pan, Wei Li, Cheng-Hui Li, Ping He, Haoshen Zhou

**Affiliations:** 1 Center of Energy Storage Materials & Technology, College of Engineering and Applied Sciences, Jiangsu Key Laboratory of Artificial Functional Materials, National Laboratory of Solid State Microstructures and Collaborative Innovation Center of Advanced Microstructures, Nanjing University, 210093 Nanjing, P. R. China; 2 State Key Laboratory of Coordination Chemistry, School of Chemistry and Chemical Engineering, Nanjing National Laboratory of Microstructures, Collaborative Innovation Center of Advanced Microstructures, Nanjing University, 210093 Nanjing, P. R. China

**Keywords:** Batteries, Electrocatalysis, Batteries, Batteries

## Abstract

Li–CO_2_ batteries possess exceptional advantages in using greenhouse gases to provide electrical energy. However, these batteries following Li_2_CO_3_-product route usually deliver low output voltage (<2.5 V) and energy efficiency. Besides, Li_2_CO_3_-related parasitic reactions can further degrade battery performance. Herein, we introduce a soluble binuclear copper(I) complex as the liquid catalyst to achieve Li_2_C_2_O_4_ products in Li–CO_2_ batteries. The Li–CO_2_ battery using the copper(I) complex exhibits a high electromotive voltage up to 3.38 V, an increased output voltage of 3.04 V, and an enlarged discharge capacity of 5846 mAh g^−1^. And it shows robust cyclability over 400 cycles with additional help of Ru catalyst. We reveal that the copper(I) complex can easily capture CO_2_ to form a bridged Cu(II)-oxalate adduct. Subsequently reduction of the adduct occurs during discharge. This work innovatively increases the output voltage of Li–CO_2_ batteries to higher than 3.0 V, paving a promising avenue for the design and regulation of CO_2_ conversion reactions.

## Abstract (web summary)

Li–CO_2_ batteries following Li_2_CO_3_-product route suffer from low output voltage and severe parasitic reactions. Herein, a soluble binuclear copper(I) complex is introduced as the liquid catalyst to achieve Li_2_C_2_O_4_ products in Li–CO_2_ batteries, which increases their output voltage to higher than 3.0 V.

## Introduction

Carbon capture and utilization (CCU) is gaining increasing attention in the field of CO_2_ reduction^1,2^, global warming mitigation^3,4^, and potentially future Mars migration^5,6^. Varieties of CCU technologies that can convert CO_2_ into value-added chemicals, such as methane dry reforming^7,8^, hydrogenation^9,10^, electrochemical reduction^11,12^, and photocatalytic reduction^13,14^, have been developed. In the past decade, a kind of energy storage device of Li–CO_2_ battery was proposed, offering an attractive tactic to utilize CO_2_ and produce electrical energy^15–17^. A typical Li–CO_2_ battery is composed of a lithium metal anode separated by an aprotic electrolyte from a porous CO_2_ cathode. The typical reversible reaction at the cathode involves the reduction of CO_2_ to form Li_2_CO_3_ and carbon on discharge, and the process is reversed on charge (Eq. 1). The thermodynamic equilibrium potential and specific energy are calculated to be about 2.80 V and 1876 Wh kg^−1^, respectively. The high theoretical specific energy far exceeds that of commercial Li-ion batteries, making it a potentially disruptive technology for energy storage^18–20^.

1 $$4{{{{{{\rm{Li}}}}}}}^{+}+3{{{{{{\rm{CO}}}}}}}_{2}+4{e}^{-}\leftrightarrow 2{{{{{{\rm{Li}}}}}}}_{2}{{{{{{\rm{CO}}}}}}}_{3}+{{{{{{\rm{C}}}}}}},\, {E}^{0}=2.80\,{{{{{\rm{V}}}}}}$$

However, practical Li–CO_2_ batteries usually present discharge voltages of around 2.5 V, sometimes even lower than 2.0 V in previous reports^21–23^. Generally, the quality of electrical energy is determined by the voltage supplied. An output voltage lower than 3.0 V leads to a low-quality electrical energy^24^. Obviously, the actual output voltage of Li–CO_2_ batteries is far lower than the theoretical value which is not high enough. Apart from the thermodynamic information of the reaction, the voltage that the battery can provide depends on the catalytic activity of catalysts and the transport properties of charge and mass in bulk and between phase boundaries.

**Fig. 1:**
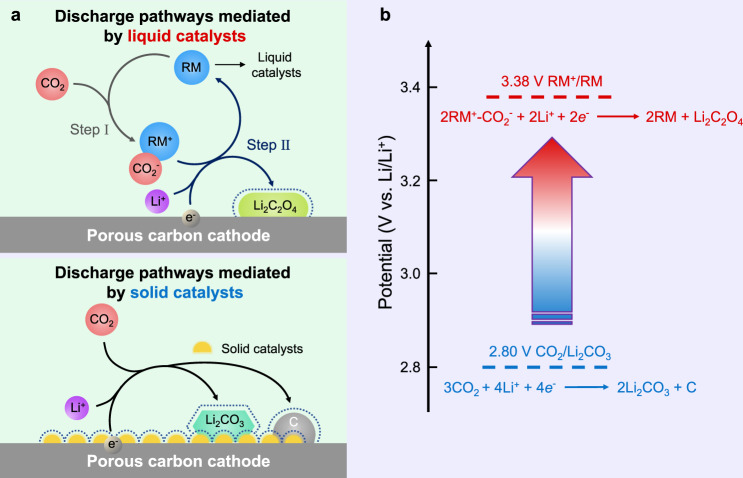
Schematic comparison of solid catalysts-mediated and liquid catalysts-mediated discharge reactions in Li–CO_2_ batteries. **a** In the presence of solid catalysts, CO_2_ is reduced to carbon and Li_2_CO_3_. Four phases are involved, including three solid phases on the cathode surface (solid catalysts, Li_2_CO_3_, and carbon) and liquid electrolyte. With the use of liquid catalysts, RM molecules capture dissolved CO_2_ in electrolyte, and then the formed RM–CO_2_ species are reduced to corresponding products and original RM. Here, the products are Li_2_C_2_O_4_. Only two phases, namely solid products and species in liquid electrolyte, are involved. **b** Discharge voltage of the battery using liquid catalyst depends on the redox potential of RM/RM^+^ couple. This condition allows to adjust the output voltage from 2.80 V (CO_2_-to-Li_2_CO_3_ conversion) to above 3.0 V by selecting and designing RM molecules.

On this basis, much efforts on solid catalysts have been exerted in raising the discharge voltage and reducing the charge voltage. The reported catalysts include carbon allotropes^25,26^, noble metals^26–28^, and transition metal oxides^29,30^. Although they can remarkably reduce the charging overpotential, they have minimal effect in increasing the discharge voltage. It should be explained here that the catalytic characteristics of solid catalysts bring the difficulty of raising battery discharge voltage. As illustrated in Fig. 1a, four phases are involved in the electrochemical reduction of CO_2_. Specifically, three solid phases contain solid catalysts, Li_2_CO_3_, and carbon on the cathode surface. The liquid phase includes Li ions and dissolved CO_2_ in electrolyte. CO_2_ reduction during discharge occurs at the catalyst/electrolyte interface. The effect of catalytic reaction partially depends on the catalytic surface area of the solid catalyst particles on which CO_2_ is reduced. The sluggish kinetics of charge-transfer and mass-transport across multiphase interfaces aggravate the large voltage hysteresis. What’s more, active sites of solid catalysts are occupied by insulating and insoluble Li_2_CO_3_ products, leading to their invalidation^31,32^.

It is worth mentioning that discharge products also affect the charging performance of the next cycle. In accordance with previous reports, electrochemical decomposition of Li_2_CO_3_ itself usually occurs irreversibly during the charging process of Li–CO_2_ batteries (Eq. 2)^33,34^. In this case, the charging potential is higher than 4.5 V and will result in the low energy efficiency. Besides, highly active intermediates of superoxide radicals (${{{{{\mathrm{O}}}}}}_{2}^{ \cdot - }$)^33^ or singlet oxygen (^1^O_2_)^34^ cause severe parasitic reactions. The gradual accumulation of irreversible byproducts threatens the stability of batteries. Thus, a catalyst designed with the strategy of Li_2_CO_3_-free pathway might be a good choice.

2 $$2{{{{{{\rm{Li}}}}}}}_{2}{{{{{{\rm{CO}}}}}}}_{3}\to 2{{{{{{\rm{CO}}}}}}}_{2}+{{{{{{\rm{O}}}}}}}_{2}^{\cdot -}({\,\!}^{1}{{{{{\rm{O}}}}}}_{2})+4{{{{{{\rm{Li}}}}}}}^{+}+3{e}^{-}$$

On the basis of the above discussion, liquid catalyst (or redox mediator, RM) rather than solid one can reduce the number of phases involved in the CO_2_ reduction process, which is effective to reduce the discharge overpotential. The reported liquid catalysts, including 2,5-ditert-butyl-1,4-benzoquinone^35^, 2-ethoxyethylamine^36^, and tris(2,2′-bipyridyl)-dichloro-ruthenium(II)^37^, can promote the discharge potential to a certain extent. However, batteries involving these catalysts still follow the Li_2_CO_3_ pathway. It might be better for the battery to discharge without taking the Li_2_CO_3_ path by liquid catalysts. As depicted in Fig. 1a, only two phases are involved. Li ions, dissolved CO_2_, and catalytic RMs are mixing at the molecular level in the liquid phase of electrolyte. Typical discharge process contains two steps. RM molecules capture CO_2_ to form RM–CO_2_ species first. The newly formed molecules then gain electrons at the cathode and are reduced to original RM and corresponding products (such as Li_2_C_2_O_4_). The liquid catalyst has more full contact with CO_2_ at the molecular level in the liquid phase, which effectively improves the reaction kinetics. The electrochemical redox process of the RM–CO_2_ at the electrode replaces the directly electrochemical reduction of CO_2_. This process allows to adjust the battery’s output voltage up to above 3.0 V by selecting and designing RM molecules (Fig. 1b).

Apart from the abovementioned conventional liquid catalysts, some soluble metal complexes can also catalyze the electrochemical reduction of CO_2_ to oxalate chemicals^38^. This condition inspires us to introduce the catalytic effect of metal complexes into the design of Li–CO_2_ batteries. Moreover, Li_2_C_2_O_4_ as an electrochemical product can take the battery out of the troublesome Li_2_CO_3_ pathway. Herein, we introduce a binuclear copper(I) complex (denoted as Cu(I) RM) as the liquid catalyst in Li–CO_2_ batteries and study the battery performance, including the discharge potential, capacity, and cycle performance, in detail. In addition, we use a variety of spectroscopic analysis techniques, such as Raman and differential electrochemical mass spectrometry (DEMS), to explore the Li_2_CO_3_-free path experienced by the discharge process of the cathode. Furthermore, we employ an additional catalyst containing Ru nanoparticles to reduce the charge overpotential synergistically. This study increases the output voltage of Li–CO_2_ batteries to more than 3.0 V, which strongly promotes the practical application of this electric energy storage system.

## Results and discussion

### Structure characterization of Cu(I) RM

The binuclear Cu(I) RM was synthesized by the reaction of disulfide ligand with two equivalents of [Cu(CH_3_CN)_4_]BF_4_ in dry acetonitrile (MeCN)^39^ (details of the preparation procedure are provided in the Methods section). ^1^H nuclear magnetic resonance spectroscopy (^1^H NMR) and electrospray ionization mass spectrometry (ESI-MS) were performed first to verify the molecular structure of the prepared ligand. As shown in Supplementary Fig. 1a, peaks at 8.53, 7.68, 7.58, 7.19, 3.80, 3.22, 2.59, and 1.25 can be observed, corresponding to H3, H5, H6, H4, H7, H10, H9, and H11 of the ligand, respectively. Besides, the signal at the mass/charge (*m/z*) ratio of 545.25 matches well with that calculated for [C_30_H_37_N_6_S_2_]^+^ of the ligand (Supplementary Fig. 1b). A yellow solution obtained was analyzed by ESI-MS after mixing the ligand with [Cu(CH_3_CN)_4_]BF_4_ in MeCN. A prominent signal at the *m/z* ratio of 335.05 in Supplementary Fig. 2 is consistent with that of [Cu_2_C_30_H_36_N_6_S_2_]^2+^, confirming the successful synthesis of the target complex.

### Electrochemical measurements of Cu(I) RM

**Fig. 2:**
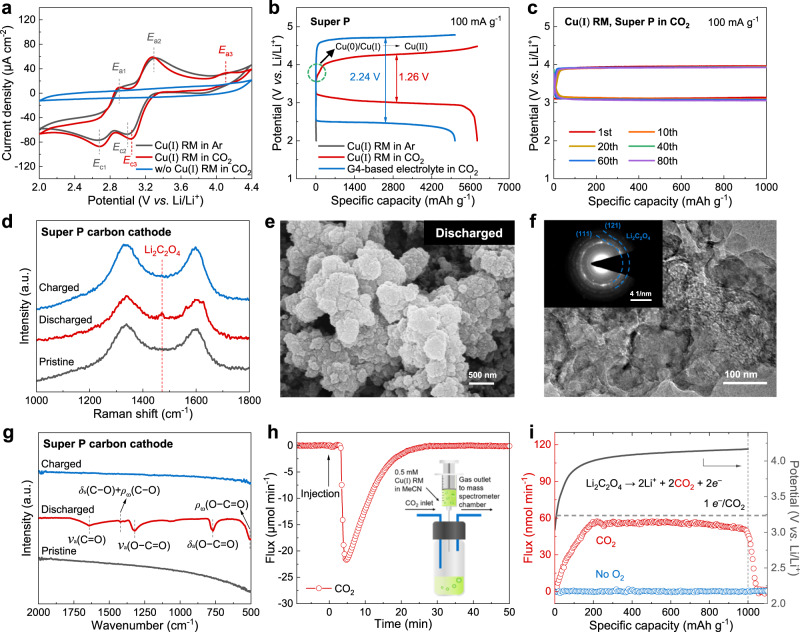
Electrochemical performance, cathode characterization, and CO_2_ quantification of Li–CO_2_ batteries with Cu(I) RM-based electrolyte. **a** CV curves for cells with various electrolytes and atmospheres: 0.5 mM Cu(I) RM in 0.1 M LiClO_4_/MeCN under Ar (dark gray), 0.5 mM Cu(I) RM in 0.1 M LiClO_4_/MeCN under CO_2_ (red), and 0.1 M LiClO_4_/MeCN under CO_2_ (blue). Scan rate is 20 mV s^−1^. **b** Galvanostatic discharge-charge curves for Super P carbon cathodes in various electrolytes and atmospheres: 0.5 mM Cu(I) RM in 0.1 M LiClO_4_/MeCN under Ar (dark gray), 0.5 mM Cu(I) RM in 0.1 M LiClO_4_/MeCN under CO_2_ (red), and 1 M LiTFSI/G4 under CO_2_ (blue). Current density is 100 mA g^−1^. **c** Cyclic performance of the Li–CO_2_ battery with Cu(I) RM-based electrolyte under a fixed specific capacity of 1000 mAh g^−1^ at a current density of 100 mA g^−1^. **d** Raman spectra of Super P carbon cathodes at different reaction stages in Li–CO_2_ batteries containing Cu(I) RM. **e** SEM and **f** TEM images of the discharged Super P carbon cathode. Corresponding SAED pattern is displayed in the inset of **f**. **g** FTIR spectra of Super P carbon cathodes at different reaction stages in Li–CO_2_ batteries containing Cu(I) RM. **h** Rate of CO_2_ consumption as a function of time after the injection of Cu(I) RM-based electrolyte. Inset of **h** shows the schematic of reaction device. Specifically, 1 mL of Cu(I) RM-based electrolyte is injected to a sealed vessel, which is connected to a mass spectrometry with pure CO_2_ as the purge gas. **i** Gas evolution rate of the Li–CO_2_ battery containing Cu(I) RM on charge. The battery is first discharged to 1000 mAh g^−1^ and subsequently charged back under the measurement of DEMS.

Electrochemical experiments were undertaken in a typical three-electrode cell with various atmospheres to investigate the catalytic effect of Cu(I) RM on CO_2_ complexation and electron transfer. Electrolyte preparation and cell assembly are described in the Methods section. As shown in Fig. 2a and Supplementary Fig. 3, two peaks appear in the cathodic region near 2.68 V (*E*_c,1_) and 2.99 V (*E*_c,2_) under Ar atmosphere. The specific redox potentials of active centers in complex molecules are affected by the structure and property of ligands^38^. Consequently, it is reasonable to assign the two cathodic peaks of *E*_c,1_ and *E*_c,2_ to the electron transfer of Cu active centers in Cu(I) RM, as depicted in Eqs. 3 and 4, respectively^40^. Meanwhile, two anodic peaks at 2.90 V (*E*_a,1_) and 3.29 V (*E*_a,2_) are rationally ascribed to reverse reactions (Fig. 2a and Supplementary Fig. 3). When CO_2_ is introduced, the cell containing Cu(I) RM displays two similar cathodic peaks to that operated in Ar, depending on the electron transfer of Cu active centers in complexes. While the cathodic peak of *E*_c,2_ moves to a more positive potential of 3.05 V (*E*_c,3_), suggesting that the reduction reaction of Cu(II) RM to Cu(I) RM is affected by the presence of CO_2_. Besides, an additional anodic peak at 4.11 V (*E*_a,3_) corresponds to the oxidation of reduction products, which will be investigated subsequently. In comparison, the CV profile shows no sign of reaction in LiClO_4_/MeCN electrolyte under CO_2_ atmosphere, which is consistent with previous reports^35^.

3 $${{{{{\rm{Cu}}}}}}({\rm I})\,{{{{{\rm{RM}}}}}}+2{e}^{-}\to {{{{{\rm{Cu}}}}}}(0)\,{{{{{\rm{RM}}}}}}$$

4 $${{{{{\rm{Cu}}}}}}({\rm I}{\rm I})\,{{{{{\rm{RM}}}}}}+2{e}^{-}\to {{{{{\rm{Cu}}}}}}({\rm I})\,{{{{{\rm{RM}}}}}}$$

To examine the practical electrochemical performance of Cu(I) RM in Li–CO_2_ batteries, galvanostatic discharge-charge tests were performed by employing home-made Swagelok-type batteries with the addition of Cu(I) RM. No solid catalyst other than Super P carbon was used on the electrode to investigate the efficacy of Cu(I) RM liquid catalysts^28^. And pre-charged Li_x_FePO_4_ instead of Li metal was used as the anode to avoid the oxidation of anode by positively charged RM species^35^ and the crossover of CO_2_ to Li anode^41–43^. The Li_x_FePO_4_ potential versus Li/Li^+^, 3.45 V, was used to express all potentials in this work at the Li scale^44^. Galvanostatic discharge-charge measurements of batteries were performed at the current density of 100 mA g^−1^ with a discharge cutoff potential of 2 V. Besides MeCN-based electrolyte, a conventional lithium bis(trifluoromethanesulphonyl)imide/tetraethylene glycol dimethyl ether (LiTFSI/G4) electrolyte was also evaluated in the Li–CO_2_ battery system for comparison^26^. All batteries rested for a minimum of 8 h before discharge-charge tests. The OCV of the Li–CO_2_ battery containing Cu(I) RM was recorded in Supplementary Fig. 4. Upon CO_2_ pumping, the OCV experiences a rapid rising from 3.12 to 3.38 V. The final OCV greatly higher than that of the conventional Li–CO_2_ battery, ~2.80 V, declares the change in battery reaction after the addition of Cu(I) RM. In the absence of Cu(I) RM, the Li–CO_2_ battery deteriorates quickly, exhibiting a small capacity of 19 mAh g^−1^ (Supplementary Fig. 5), which is consistent with the ignorable current response measured by CV test (Fig. 2a). As presented in Fig. 2b, on the one hand, the Li–CO_2_ battery containing Cu(I) RM catalyst delivers a larger discharge capacity up to 5846 mAh g^−1^ and a higher output voltage of about 3.04 V, indicating the great effect of Cu(I) RM on the promotion of discharge performance. On the other hand, the battery shows a reduced charge voltage plateau of 4.27 V compared with those without Cu(I) RM. As is known to all, the conventional Li–CO_2_ battery usually exhibits poor rechargeability and low energy efficiency^45^. The decrease in charge potential could be related to Cu(I) RM-mediated discharge reaction path. Notably, a short slope at the beginning of charging in the battery containing Cu(I) RM can be connected with the oxidation of Cu(0)/Cu(I) to Cu(II) after deep discharge. As shown in Supplementary Fig. 6, the deconvoluted Cu 2p spectrum of discharged Super P carbon cathode shows peaks assigned to Cu^0^/Cu^+^ and Cu^2+^^46^. By contrast, the battery containing Cu(I) RM catalyst has almost no capacity when the CO_2_ was replaced by Ar, indicating that the discharge capacity is associated with participation of CO_2_ in the reaction. In the absence of CO_2_, only the reduction of Cu(I) RM to Cu(0) RM occurs. The small addition of Cu(I) RM in battery accounts for its very small capacity. Furthermore, under a fixed specific capacity of 1000 mAh g^−1^, the Li–CO_2_ battery with Cu(I) RM exhibits steady cyclability over 80 cycles (Fig. 2c).

### Cu(I) RM-mediated reaction mechanism

In order to unveil the specific discharge product of Li–CO_2_ batteries in the presence of Cu(I) RM, the morphology and composition of Super P carbon cathodes at different reaction stages, including pristine, after discharge, and after recharge, were characterized. Supplementary Fig. 7a shows the typical morphology of Super P carbon particles. Figure 2d presents Raman peaks corresponding to the D and G bands of Super P carbon at 1338 and 1600 cm^−1^, respectively. After discharge, randomly arranged products are deposited densely on the surface of Super P carbon cathode (Fig. 2e). Transmission electron microscopy (TEM) image clearly manifests that Super P carbon particles are well-coated with discharge products (Fig. 2f). The corresponding selected area electron diffraction (SAED) pattern indicates that these products are Li_2_C_2_O_4_. Besides, a new peak at 1473 cm^−1^ assigned to Li_2_C_2_O_4_ is observed for the discharged Super P carbon electrode in the Raman spectrum^47^ (Fig. 2d). In addition, Fourier transform infrared (FTIR) spectrum displays peaks at 508, 771, 1321, 1422, and 1641 cm^−1^, which are indexed to *ρ*_ω_(O−C=O), *δ*_a_(O−C=O), *ν*_a_(O−C=O), *δ*_s_(C−O) + *ρ*_ω_(C−O), and *ν*_a_(C=O) modes of C_2_O_4_^2−^, respectively^48–51^ (Fig. 2g). And the characteristic peak of O−C=O for Li_2_C_2_O_4_ is detected at 288.95 eV in the C 1 s spectrum through X-ray photoelectron spectroscopy (XPS)^43^ (Supplementary Fig. 8). It is clearly evidenced that the discharge product is Li_2_C_2_O_4_ rather than Li_2_CO_3_. Thus, the formation of Li_2_C_2_O_4_ products accounts for the reduced charge voltage shown in Fig. 2b. When the charge process is finished, these discharged particles disappear, and the cathode surface is recovered (Supplementary Fig. 7b). Meanwhile, all the peaks assigned to Li_2_C_2_O_4_ vanish, confirming the fully reversible decomposition of Li_2_C_2_O_4_ products after recharging (Fig. 2d, g). The reversible formation and decomposition of Li_2_C_2_O_4_ products can be detected even after multiple cycles (Supplementary Fig. 9).

Subsequently, mass spectrometry was conducted to analyze the CO_2_ gas consumed by Cu(I) RM, and in situ DEMS was performed to reveal the ratio of transferred electrons and generated CO_2_ gas during charge. As shown in Fig. 2h, a sealed vessel was connected to the quadrupole mass spectrometry with pure CO_2_ gas stream as the purge gas. The Cu(I) RM-based electrolyte was injected into the vessel when the flux of CO_2_ reached a stable background line. The flux of CO_2_ declines sharply in the first 5 min and increases afterwards, demonstrating the rapid reaction kinetics between Cu(I) RM and CO_2_. After discharged to a limited specific capacity of 1000 mAh g^−1^, the Cu(I) RM-contained Li–CO_2_ battery was charged back and monitored by DEMS. No gases were detected other than CO_2_ (Fig. 2i). The evolution of CO_2_ increases gradually and stabilizes with a charge-to-mass ratio close to 1 *e*^–^/CO_2_, verifying Li_2_C_2_O_4_ decomposition as the dominant charge reaction (Eq. 5).

5 $${{{{{{\rm{Li}}}}}}}_{2}{{{{{{\rm{C}}}}}}}_{2}{{{{{{\rm{O}}}}}}}_{4}\to 2{{{{{{\rm{Li}}}}}}}^{+}+2{{{{{{\rm{CO}}}}}}}_{2}+2{e}^{-}$$

**Fig. 3:**
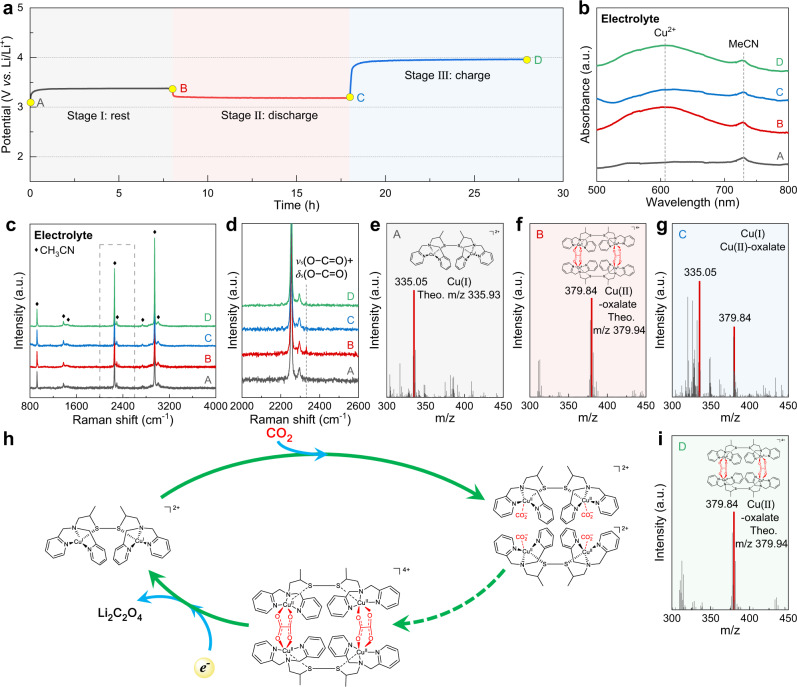
Electrolyte characterization of Li–CO_2_ batteries with Cu(I) RM-based electrolyte. **a** Discharge-charge curve of the Li–CO_2_ battery with the addition of Cu(I) RM, showing three stages, namely rest, discharge, and charge. **b** UV–Vis and **c** Raman spectra of Cu(I) RM-based electrolytes in the Li–CO_2_ battery at different reaction stages. **d** shows the enlarged view of **c**. ESI-MS spectra of Cu(I) RM-based electrolytes at different reaction stages, showing **e** pristine, **f** before discharge, and **g** after discharge. **h** Proposed mechanism of Cu(I) RM-mediated CO_2_ reduction process. **i** ESI-MS spectrum of Cu(I) RM-based electrolyte after recharge.

To further elucidate changes in the chemical structures for Cu(I) RM molecules at different electrochemical states, Cu(I) RM-based electrolytes corresponding to different reaction stages in Fig. 3a were explored by ultraviolet–visible (UV–Vis) absorption spectroscopy, Raman spectroscopy, and ESI-MS. No particular features but signals associated with Cu(I) RM and MeCN solvent can be observed for the pristine electrolyte (Fig. 3b–e). A broad band centered at 606.70 nm arises in the UV–Vis spectrum while resting in CO_2_ for a period (Fig. 3b). The absorbance band matches well with the d–d transition of Cu^2+^ center in the bridged CO_2_-derived oxalate group^52^. And a new peak at 2332 cm^−1^ referring to the vibration mode of *ν*_s_(O−C=O) + *δ*_s_(O−C=O) in oxalate group is recorded in the Raman spectrum^49^ (Fig. 3c, d). Besides, a prominent signal at the *m/z* of 379.84 in Fig. 3f is consistent with that of the bridged Cu(II)-oxalate adduct ([Cu_4_C_64_H_72_N_12_O_8_S_4_]^4+^)^39^. These results indicate that Cu(I) RM molecules capture CO_2_ in the electrolyte to form the Cu(II)-oxalate adduct primarily. As calculated by the integration of CO_2_ consumption rate and time in Fig. 2h, the total amount of CO_2_ consumed is equal to the summation of reacting CO_2_ with Cu(I) RM molecules and dissolved CO_2_ in MeCN^53^, confirming that the Cu(II)-oxalate adduct is generated by the reaction of two equivalents of CO_2_ to one Cu(I) RM, as described in Eq. 6. All the characteristic peaks ascribed to Cu(II)-oxalate adduct decrease obviously, and the signal of Cu(I) RM in Fig. 3g appears again after discharge, indicating the regeneration of Cu(I) RM with the formation of Li_2_C_2_O_4_ (Eq. 7). Thus, redox mediation by the Cu(I) RM involves a complexation mechanism and subsequent electron transport (Fig. 3h). The formation of Cu(II)-oxalate adduct can be caused by the rapid reaction between Cu(I) RM and dissolved CO_2_ in the electrolyte, as evidenced by mass spectrometry (Fig. 2h). After recharge, apart from features of Cu(II)-oxalate adduct (Fig. 3i), a signal consistent with that calculated for the electrochemically oxidized Cu(I) RM ([Cu_2_C_30_H_36_N_6_S_2_]^4+^) is observed (Supplementary Fig. 10). As presented in Supplementary Fig. 11, Cu(I) RM-based electrolyte can remain stable after multiple cycles.

6 $$2{{{{{\rm{Cu}}}}}}({\rm I})\,{{{{{\rm{RM}}}}}}+4{{{{{{\rm{CO}}}}}}}_{2}\to {{{{{\rm{Cu}}}}}}({\rm I}{\rm I})\mbox{-}{({{{{{{\rm{C}}}}}}}_{2}{{{{{{\rm{O}}}}}}}_{4})}_{2}$$

7 $${{{{{\rm{Cu}}}}}}({\rm I}{\rm I})\mbox{-}{({{{{{{\rm{C}}}}}}}_{2}{{{{{{\rm{O}}}}}}}_{4})}_{2}+4{{{{{{\rm{Li}}}}}}}^{+}+4{e}^{-}\to 2{{{{{\rm{Cu}}}}}}({\rm I})\,{{{{{\rm{RM}}}}}}+2{{{{{{\rm{Li}}}}}}}_{2}{{{{{{\rm{C}}}}}}}_{2}{{{{{{\rm{O}}}}}}}_{4}$$

On the basis of the above results, Li–CO_2_ electrochemistry mediated by Cu(I) RM is clear. That is, Cu(I) RM reacts with CO_2_ to form Cu(II)-oxalate adduct chemically first, then the newly formed adduct gets reduced electrochemically to produce Li_2_C_2_O_4_ product and original Cu(I) RM. Other two possible pathways are also taken into consideration^54^. One path is that Cu(I) RM gets reduced electrochemically to form Cu(0) RM first, then Cu(0) RM reacts with CO_2_ chemically. As shown in Fig. 2a, the reduction peak of Cu(I) RM to Cu(0) RM is about 2.68 V (*E*_c,1_), much lower than the practical discharge voltage plateau of 3.04 V in this RM-involved Li–CO_2_ battery. Thus, this path can be ruled out. Another path is that CO_2_ gets reduced electrochemically to form CO_2_^−^ first, then CO_2_^−^ reacts with Cu(I) RM chemically. Considering the low thermodynamic potential of CO_2_/CO_2_^−^ conversion (−1.9 V versus NHE)^55^, the discharge voltage of a Li–CO_2_ battery following this path should be no more than 2.0 V versus Li/Li^+^. In consequence, this path can also be ruled out.

Obviously, the Cu(I) RM-mediated reaction process of Li–CO_2_ battery possesses some unique advantages. Firstly, the Cu(I) RM can greatly promote the output voltage of battery to above 3.0 V by converting the direct electrochemical reduction of CO_2_ into the reduction of bridged Cu(II)-oxalate adduct. Then, the Cu(I) RM can increase the discharge capacity of battery significantly until the cathode is covered by enough solid products. Besides, Li_2_C_2_O_4_ products are formed rather than troublesome Li_2_CO_3_, leading to a relatively lower charge platform. In addition, aggressive intermediates derived from Li_2_CO_3_ decomposition are circumvented, thereby endowing a prolonged cycle life of more than 80 cycles. However, the charge potential plateau beyond 4.0 V is still unsatisfactory. As we know, Super P carbon generally exhibits poor electrocatalytic activity. Thus, more effective catalysts are required to further improve the charge performance of the Cu(I) RM-containing Li–CO_2_ battery.

### Synergistic effect of Cu(I) RM and Ru catalysts

**Fig. 4:**
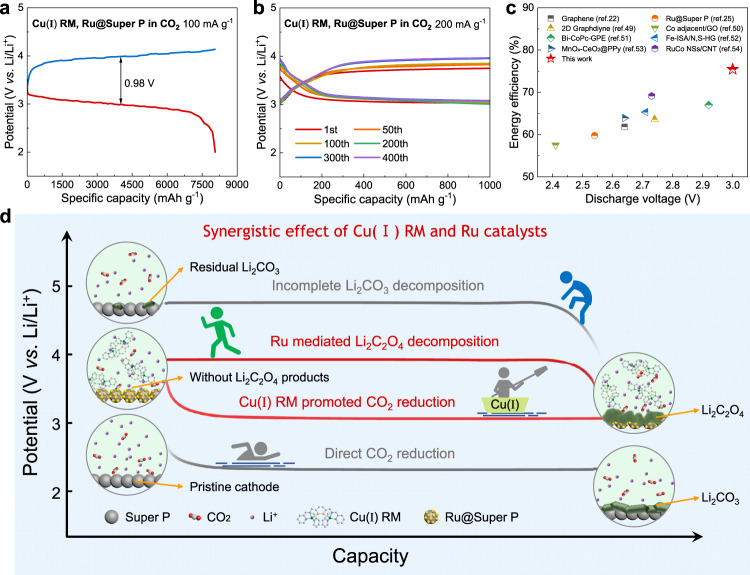
Optimization of charge performance by Ru catalysts. **a** Galvanostatic discharge-charge curves of the Li–CO_2_ battery with a Cu(I) RM-based electrolyte and a Ru@Super P cathode during deep discharging at a current density of 100 mA g^−1^. **b** Cyclic performance of the Li–CO_2_ battery with a Cu(I) RM-based electrolyte and a Ru@Super P cathode under a fixed specific capacity of 1000 mAh g^−1^ at a current density of 200 mA g^−1^. **c** Comparison of discharge voltage and energy efficiency with other reported catalysts for Li–CO_2_ batteries in recent reports ^25,28,56–61^. **d** Schematic of the synergetic effect of Cu(I) RM and Ru catalysts for the Li–CO_2_ battery system.

Considering the excellent catalytic properties of Ru in reducing charge overpotential^27,28^, Ru nanoparticles deposited on Super P carbon (Ru@Super P) were incorporated as the cathode catalyst in this Cu(I) RM-involved Li–CO_2_ battery. The preparation procedure of Ru@Super P is depicted in the Methods section. All peaks in the X-ray diffraction (XRD) pattern of Ru@Super P can be assigned to metallic Ru (Supplementary Fig. 12a). The TEM image clearly exhibits that Ru nanoparticles are well-dispersed on Super P carbon (Supplementary Fig. 12b). Additionally, the Ru content in Ru@Super P was estimated to be around 17% according to thermogravimetric (TG) analysis (Supplementary Fig. 12c). To demonstrate the stability of Cu(I) RM in the presence of Ru catalyst, the Ru@Super P powder was immersed in the Cu(I) RM-based electrolyte for at least 10 days. It can be seen from Supplementary Fig. 13 that the prominent signal at the *m/z* ratio of 335.05 consistent with that of Cu(I) RM can also be detected in the ESI-MS spectrum, indicating that the application of Ru catalyst has no effect on the stability of Cu(I) RM. Figure 4a displays the galvanostatic discharge-charge curve of the Li–CO_2_ battery with Cu(I) RM-based electrolyte and Ru@Super P cathode at the current density of 100 mA g^−1^. The discharge plateau of around 3.01 V is close to that using the Super P carbon cathode, and an enlarged specific discharge capacity of about 8058 mAh g^−1^ is obtained. More importantly, the charge platform is remarkably decreased to 3.99 V, and the energy efficiency is enhanced to 75.4%, demonstrating the excellent catalytic activity of Ru nanoparticles toward the charging process. Notably, the Ru catalyst has no effect on the Cu(I) RM-mediated reaction path as Li_2_C_2_O_4_ was detected as the sole discharge product (Supplementary Fig. 14). Moreover, the cycling performance of the battery was assessed with a limited specific capacity of 1000 mAh g^−1^ at a current density of 200 mA g^−1^. As shown in Fig. 4b, the battery exhibits robust cycle stability over 400 cycles. The discharge voltage (3.01 V) and energy efficiency (75.4%) of this work are the best results among the reported solid or liquid catalysts during full discharge-recharge process at the current density of 100 mA g^−1^ ^25,28,56–61^ (Fig. 4c). The highly stable and catalytic Ru nanoparticles play an important role in the reversible decomposition of Li_2_C_2_O_4_ products during multiple cycles (Supplementary Fig. 15). Figure 4d summarizes the synergistic effect of soluble Cu(I) RM and solid Ru catalysts in the Li–CO_2_ battery system. Specifically, Cu(I) RM acting as a molecular shuttle of CO_2_ manipulates the discharge path and gains Li_2_C_2_O_4_ products through a liquid-liquid catalysis route; Ru nanoparticles provide highly catalytic active sites for Li_2_C_2_O_4_ decomposition on charge. Thus, a high discharge voltage above 3.0 V and a small discharge-charge voltage gap within 1.0 V are obtained, enabling the significantly increased energy efficiency.

In conclusion, we introduced Cu(I) RM as the liquid catalyst to achieve Li_2_C_2_O_4_ products in Li–CO_2_ batteries. Specifically, the Li–CO_2_ battery with Cu(I) RM exhibits a remarkably promoted electromotive voltage up to 3.38 V that far exceeds those of other reported Li–CO_2_ batteries. Electrochemical and spectroscopic investigations clearly reveal the reaction route of Li–CO_2_ batteries involving Cu(I) RM: Firstly, the Cu(I) RM captures CO_2_ to form a bridged Cu(II)-oxalate adduct; Secondly, the formed adduct gets reduced with the formation of original Cu(I) RM and Li_2_C_2_O_4_ products during discharge; Finally, Li_2_C_2_O_4_ products are decomposed in the charge process. With the addition of Cu(I) RM, the Li–CO_2_ battery using the Super P carbon cathode delivers an enhanced discharge plateau of about 3.04 V, an enlarged discharge capacity of 5846 mAh g^−1^, and a reduced charge voltage plateau of around 4.27 V. Besides, under the fixed capacity of 1000 mAh g^−1^, the Li–CO_2_ battery exhibits steady cyclability over 80 cycles. Then, the Ru@super P catalysts are employed to further reduce the charging polarization. Benefiting from the synergistic effect of soluble Cu(I) RM and solid Ru catalysts, a similar discharge potential of 3.01 V, a promoted discharge capacity of 8058 mAh g^−1^, and a remarkably decreased charge potential of about 3.99 V are achieved. Under the fixed specific capacity of 1000 mAh g^−1^, the battery shows robust cycle stability over 400 cycles. This study increases the output voltage of Li–CO_2_ batteries to higher than 3.0 V and provides convictive evidence of the Li_2_CO_3_-free discharge route utilizing an effective soluble metal complex, thereby offering an approach to improving the electrochemical performance of Li–CO_2_ batteries as well as propelling their practical application.

## Methods

### Materials

All chemicals used in the synthesis were purchased from Sigma–Aldrich or Tokyo Chemical Industry Co., Ltd. without further purification. For the components in the electrolytes, LiClO_4_ was obtained from Alfa Aesar. LiTFSI, G4, and MeCN were obtained from Sigma–Aldrich. The salts were dried under vacuum at 120 °C for 12 h, and solvents were dried by using activated 4 Å molecular sieves prior to use.

### Preparation of Cu(I) RM

The binuclear Cu(I) RM was synthesized as described in previous literature^39^. Firstly, dipicolylamine (1.196 g, 6 mmol) and propylene sulfide (0.445 g, 6 mmol) were added to ultra-dry MeCN of 10 mL and experienced reflux under Ar atmosphere for 12 h. Then, the solution was stirred in air for 12 h at room temperature. After that, the solvent was evaporated under vacuum to obtain the light yellow oily ligand. Finally, [Cu(CH_3_CN)_4_]BF_4_ (0.3 g, 1 mmol) was added to the MeCN solution containing ligand (0.27 g, 0.5 mmol) in an Ar glove box to acquire the Cu(I) RM.

### Preparation of Ru@Super P

Ru@Super P was prepared in accordance with our previous study^28^: RuCl_3_•*x*H_2_O (50 mg) was dissolved in ethylene glycol of 100 mL. Then Super P carbon (80 mg) was added in the solution, where the suspension was stirred for 3 h at 170 °C via an oil bath. The mixtures were filtered by deionized water and ethanol for several times after cooling to room temperature. The final products were dried at 120 °C under vacuum for 12 h.

### Characterization of Cu(I) RM

All samples were transferred to different characterization equipment by using an air-tight sample module. ^1^H NMR (Bruker DRX500) was applied to analyze the molecular structures of the ligand. ESI-MS (Agilent 6460) was performed to collect information of the ligand and Cu(I) RM.

### Battery assembly and electrochemical performance tests

A three-electrode glass cell was first used to conduct CV tests. The working electrode was commercial glassy carbon (GC, *Φ* 3 mm) that was polished elaborately prior to use. The counter electrode was obtained by rolling the mixture of LiFePO_4_, Super P carbon, and polytetrafluoroethylene (PTFE) binder (W: W: W = 80: 10: 10) into a film (1.0 × 1.2 cm) and pressing on a stainless steel (SS) current collector. The pre-charged (10% of total capacity) Li_x_FePO_4_ (x = 0.9) electrode was applied as the reference electrode, which had a stable potential of 3.45 V versus Li/Li^+^. The galvanostatic discharge-charge measurements were conducted in Swagelok batteries, including a Super P carbon (or Ru@Super P) cathode (*Φ* 12 mm, 1.13 cm^2^), a pre-charged Li_x_FePO_4_ anode (*Φ* 12 mm), a glassy fiber separator (*Φ* 12 mm, Whatman), and a gas chamber. The cathode was prepared by rolling the mixture of Super P carbon (or Ru@Super P) and PTFE binder (W: W = 85: 15) into a film and pressing on a SS mesh. The mass loading of the electrode was 0.5 ± 0.2 mg cm^−2^. The thickness of a glassy fiber separator was 675 μm. All the electrodes were dried at 120 °C under vacuum for at least 12 h before assemblage. LiClO_4_ (0.1 M) in MeCN with or without Cu(I) RM (0.5 mM) was employed as electrolytes and the amount of electrolyte in each battery was about 300 μL.

CV measurements were carried out on an electrochemical workstation (CHI760E, Chenhua Co., Shanghai, P. R. China) at 25 °C inside an Ar or CO_2_-filled glove box with a pressure of 1 atm, as well as negligible H_2_O and O_2_ levels (<0.1 ppm). Galvanostatic tests were performed on LAND 2001A Battery Testing Systems (Wuhan LAND electronics Co., Ltd, P. R. China) at 25 °C under Ar or CO_2_ atmosphere. The batteries were discharged and charged at a specific current of 100 mA g^−1^ and potential cut-offs of 2 V and 4.8 V. Galvanostatic discharge/charge cycling tests were conducted at a constant current density of 100 mA g^−1^ or 200 mA g^−1^ and a fixed capacity of 1000 mAh g^−1^. All current densities and capacities were normalized by the mass of active materials on the cathode. The specific energy based on active substance on the cathode was the product of specific capacity and output voltage.

### Electrode and electrolyte characterization

The discharged and recharged electrodes were washed with MeCN and dried sufficiently before characterization. The components of electrodes and electrolytes during different reaction stages were recorded by Raman spectroscopy (Renishaw inVia confocal Raman microscope) with the excitation light of an air-cooled He–Ne laser at 633 nm through a 50× long working distance lens (Leica Microsystems Inc.). To obtain apparent signals on the spectra and avoid the degradation of carbon cathode, the acquisition time was set as 120 s with 10% laser power. The resolution of Raman spectroscopy was around 1.0 cm^−1^. FTIR measurement was conducted on a FTIR spectroscope (PerkinElmer, Spectrum Two LiTa) with a wavenumber range of 4000–450 cm^−1^ and a resolution of 1.0 cm^−1^. The states of surface elements on the cathodes were characterized through XPS (Thermo Fisher Scientific Model K-Alpha spectrometer) equipped with Al Ka radiation (1486.6 eV) at a working voltage of 12 kV and a current of 10 mA. The morphology of cathodes was observed by SEM (Hitachi SU8010). The microstructure was further characterized by TEM (FEI TF20), and the SAED pattern was collected from a Gatan charge-coupled device camera. To evaluate the interaction between Cu(I) RM and CO_2_ in the electrolyte, ESI-MS and UV–vis absorption spectra data were collected. The UV–vis spectra were evaluated on a UV–vis spectrophotometer (Beijing Purkinje General Instrument Co. Ltd., P. R. China). XRD analysis was performed to analyze the crystalline structure of the catalyst by employing a Bruker D8 advanced diffractometer with Cu–Kα radiation (*λ* = 1.5406 Å) at a scan rate of 0.064° s^−1^. TG was carried out on an SDT Q600 TA instrument with a temperature range of 25–800 °C in O_2_ gas and the heating rate was 5 °C min^−1^.

### Differential electrochemical mass spectrometry characterization

In situ DEMS measurements were performed by chemical/electrochemical reactions. With regard to the chemical reaction, a mixture of CO_2_/Ar (V: V = 9: 1) was purged continuously to eliminate residual air first, after which 0.5 mM Cu(I) RM in MeCN (1 mL) was injected to the sealed vessel, and the remaining gas was purged to the mass spectrometer chamber (PrismaPro QMG 250 M2). The sealed vessel was connected by using two PEEK valves to the purge gas system. The electrochemical reaction was conducted by a home-made Li–CO_2_ battery mold with two PEEK valves connected to a quadrupole mass spectrometer with a turbomolecular pump (Pfeiffer Vacuum). During the charge process, ultrapure Ar was employed as carrier with a flux of 0.5 mL min^−1^. The DEMS battery was also performed on LAND 2001A Battery Testing Systems.

## Supplementary information

Supplementary Information

## Data Availability

The data generated in this study are provided in the paper and Supplementary Information. Additional relevant data are available from the corresponding author on request.

## References

[1] Fankhauser S (2021). The meaning of net zero and how to get it right. Nat. Clim. Change.

[2] Nakano K, Hoshino Y, Numata K, Tanaka K (2021). Special issue: CO_2_: capture of, utilization of, and degradation into. Polym. J..

[3] Mac Dowell N, Fennell PS, Shah N, Maitland GC (2017). The role of CO_2_ capture and utilization in mitigating climate change. Nat. Clim. Change.

[4] Friedlingstein P (2014). Persistent growth of CO_2_ emissions and implications for reaching climate targets. Nat. Geosci..

[5] Jakosky BM, Edwards CS (2018). Inventory of CO_2_ available for terraforming Mars. Nat. Astron..

[6] Steele A (2018). Organic synthesis on Mars by electrochemical reduction of CO_2_. Sci. Adv..

[7] Akri M (2019). Atomically dispersed nickel as coke-resistant active sites for methane dry reforming. Nat. Commun..

[8] Palmer C (2020). Dry reforming of methane catalysed by molten metal alloys. Nat. Catal..

[9] Ye R-P (2019). CO_2_ hydrogenation to high-value products via heterogeneous catalysis. Nat. Commun..

[10] Zhou H (2021). Engineering the Cu/Mo_2_CT_x_ (MXene) interface to drive CO_2_ hydrogenation to methanol. Nat. Catal..

[11] Sullivan I (2021). Coupling electrochemical CO_2_ conversion with CO_2_ capture. Nat. Catal..

[12] Wakerley D (2022). Gas diffusion electrodes, reactor designs and key metrics of low-temperature CO_2_ electrolysers. Nat. Energy.

[13] Ulmer U (2019). Fundamentals and applications of photocatalytic CO_2_ methanation. Nat. Commun..

[14] Gao W (2020). Industrial carbon dioxide capture and utilization: state of the art and future challenges. Chem. Soc. Rev..

[15] Xu S, Das SK, Archer LA (2013). The Li–CO_2_ battery: a novel method for CO_2_ capture and utilization. RSC Adv..

[16] Guan DH (2020). Light/electricity energy conversion and storage for a hierarchical porous In_2_S_3_@CNT/SS cathode towards a flexible Li–CO_2_ battery. Angew. Chem. Int. Ed. Engl..

[17] Wang XX (2021). A renewable light-promoted flexible Li–CO_2_ battery with ultrahigh energy efficiency of 97.9%. Small.

[18] Mu X, Pan H, He P, Zhou H (2020). Li–CO_2_ and Na–CO_2_ batteries: toward greener and sustainable electrical energy storage. Adv. Mater..

[19] Xie Z, Zhang X, Zhang Z, Zhou Z (2017). Metal–CO_2_ Batteries on the road: CO_2_ from contamination gas to energy source. Adv. Mater..

[20] Guan DH (2022). All-solid-state photo-assisted Li–CO_2_ battery working at an ultra-wide operation temperature. ACS Nano.

[21] Zhang Z, Bai W-L, Wang K-X, Chen J-S (2020). Electrocatalyst design for aprotic Li–CO_2_ batteries. Energy Environ. Sci..

[22] Iputera K (2022). Revealing the absence of carbon in aprotic Li–CO_2_ batteries: a mechanism study toward CO_2_ reduction under a pure CO_2_ environment. J. Mater. Chem. A.

[23] Xu S (2018). Flexible lithium–CO_2_ battery with ultrahigh capacity and stable cycling. Energy Environ. Sci..

[24] Huggins, R. A. Principles determining the voltages and capacities of electrochemical cells. *Energy Storage* 145–160 (2016).

[25] Zhang Z (2015). The first introduction of graphene to rechargeable Li–CO_2_ batteries. Angew. Chem. Int. Ed. Engl..

[26] Chen B (2021). Engineering the active sites of graphene catalyst: from CO_2_ activation to activate Li–CO_2_ batteries. ACS Nano.

[27] Qiao Y (2019). Transient, in situ synthesis of ultrafine ruthenium nanoparticles for a high-rate Li–CO_2_ battery. Energy Environ. Sci..

[28] Yang S (2017). A reversible lithium–CO_2_ battery with Ru nanoparticles as a cathode catalyst. Energy Environ. Sci..

[29] Li S (2019). Monodispersed MnO nanoparticles in graphene-an interconnected N-doped 3D carbon framework as a highly efficient gas cathode in Li–CO_2_ batteries. Energy Environ. Sci..

[30] Zhuo Z (2021). Cycling mechanism of Li_2_MnO_3_: Li–CO_2_ batteries and commonality on oxygen redox in cathode materials. Joule.

[31] Garcia-Lastra JM, Myrdal JSG, Christensen R, Thygesen KS, Vegge T (2013). DFT+U study of polaronic conduction in Li_2_O_2_ and Li_2_CO_3_: implications for Li–air batteries. J. Phys. Chem. C..

[32] Ling C, Zhang R, Takechi K, Mizuno F (2014). Intrinsic barrier to electrochemically decompose Li_2_CO_3_ and LiOH. J. Phys. Chem. C..

[33] Yang S, He P, Zhou H (2016). Exploring the electrochemical reaction mechanism of carbonate oxidation in Li–air/CO_2_ battery through tracing missing oxygen. Energy Environ. Sci..

[34] Mahne N, Renfrew SE, McCloskey BD, Freunberger SA (2018). Electrochemical oxidation of lithium carbonate generates singlet oxygen. Angew. Chem. Int. Ed. Engl..

[35] Yin W, Grimaud A, Azcarate I, Yang C, Tarascon J-M (2018). Electrochemical reduction of CO_2_ mediated by quinone derivatives: implication for Li–CO_2_ battery. J. Phys. Chem. C..

[36] Khurram A, He M, Gallant BM (2018). Tailoring the discharge reaction in Li–CO_2_ batteries through incorporation of CO_2_ capture chemistry. Joule.

[37] Zhang Z (2021). Enhanced electrochemical performance of aprotic Li–CO_2_ batteries with a ruthenium-complex-based mobile catalyst. Angew. Chem. Int. Ed. Engl..

[38] Takeda H, Cometto C, Ishitani O, Robert M (2017). Electrons, photons, protons and earth-abundant metal complexes for molecular catalysis of CO_2_ reduction. ACS Catal..

[39] Angamuthu R, Byers P, Lutz M, Spek Anthony L, Bouwman E (2010). Electrocatalytic CO_2_ conversion to oxalate by a copper complex. Science.

[40] Deng H (2019). Killing two birds with one stone: a Cu ion redox mediator for a non-aqueous Li–O_2_ battery. J. Mater. Chem. A.

[41] Bharti A, Manna G, Saha P, Achutarao G, Bhattacharyya AJ (2022). Probing the function of a Li-CO_2_ battery with a MXene/graphene oxide composite cathode electrocatalyst. J. Phys. Chem. Lett..

[42] He P (2020). Towards a stable Li–CO_2_ battery: the effects of CO_2_ to the Li metal anode. Energy Stor. Mater..

[43] Feng N (2021). Mechanism-of-action elucidation of reversible Li–CO_2_ batteries using the water-in-salt electrolyte. ACS Appl. Mater. Interfaces.

[44] Gao X, Chen Y, Johnson L, Bruce PG (2016). Promoting solution phase discharge in Li–O_2_ batteries containing weakly solvating electrolyte solutions. Nat. Mater..

[45] Sun X, Hou Z, He P, Zhou H (2021). Recent advances in rechargeable Li–CO_2_ batteries. Energy Fuels.

[46] Ivanova TM (2020). XPS detection of unusual Cu(II) to Cu(I) transition on the surface of complexes with redox-active ligands. J. Electron Spectrosc..

[47] Edwards HGM, Lewis IR (1994). FT-Raman spectroscopic studies of metal oxalates and their mixtures. Spectrochim. Acta A Mol. Spectrosc..

[48] Fujita J, Nakamoto K, Kobayashi M (1957). Infrared spectra of metallic complexes. III. the infrared spectra of metallic oxalates. J. Phys. Chem..

[49] Clark RJH, Firth S (2002). Raman, infrared and force field studies of K_2_^12^C_2_O_4_ · H_2_O and K_2_^13^C_2_O_4_ · H_2_O in the solid state and in aqueous solution, and of (NH_4_)_2_^12^C_2_O_4_ · H_2_O and (NH_4_)_2_^13^C_2_O_4_ · H_2_O in the solid state. Spectrochim. Acta A Mol. Biomol. Spectrosc..

[50] Socrates, G. *Infrared and Raman Characteristic Group Frequencies: Tables and Charts*. (Wiley Chichester, 2002).

[51] Nakamoto, K. *Infrared and Raman Spectra of Inorganic and Coordination Compounds: Part B: Applications in Coordination, Organometallic, and Bioinorganic Chemistry*. (John Wiley & Sons, 2008).

[52] Maalej W, Guionneau P, Elaoud Z (2021). A new square pyramidal copper(II) complex [Cu(C_10_H_24_N_4_)Br]Br: crystal structure, thermal analysis, hirschfeld surfaces, electrical and semiconducting properties. J. Mol. Struct..

[53] Rudnev AV (2016). The promoting effect of water on the electroreduction of CO_2_ in acetonitrile. Electrochim. Acta.

[54] Mandal S, Samajdar RN, Parida S, Mishra S, Bhattacharyya AJ (2022). Transition metal phthalocyanines as redox mediators in Li–O_2_ batteries: a combined experimental and theoretical study of the influence of 3d electrons in redox mediation. ACS Appl. Mater. Interfaces.

[55] Benson EE, Kubiak CP, Sathrum AJ, Smieja JM (2009). Electrocatalytic and homogeneous approaches to conversion of CO_2_ to liquid fuels. Chem. Soc. Rev..

[56] Zhang J (2021). Rechargeable Li–CO_2_ batteries with graphdiyne as efficient metal‐free cathode catalysts. Adv. Funct. Mater..

[57] Zhang BW (2019). Targeted synergy between adjacent Co atoms on graphene oxide as an efficient new electrocatalyst for Li–CO_2_ batteries. Adv. Funct. Mater..

[58] Li J (2019). Drawing a pencil-trace cathode for a high-performance polymer-based Li–CO_2_ battery with redox mediator. Adv. Funct. Mater..

[59] Hu C (2020). High-performance, long-life, rechargeable Li–CO_2_ batteries based on a 3D holey graphene cathode implanted with single iron atoms. Adv. Mater..

[60] Deng Q (2022). Electronic state modulation and reaction pathway regulation on necklace‐like MnO_x_‐CeO_2_@polypyrrole hierarchical cathode for advanced and flexible Li–CO_2_ batteries. Adv. Energy Mater..

[61] Wang Y (2022). Decreasing the overpotential of aprotic Li–CO_2_ batteries with the in‐plane alloy structure in ultrathin 2D Ru‐based nanosheets. Adv. Funct. Mater..

