# Supplementary material for: Binuclear Cu complex catalysis enabling Li–CO2 battery with a high discharge voltage above 3.0 V
Source: Nat Commun. 2023 Feb 1;14:536. doi: 10.1038/s41467-023-36276-8 (PMC9892515; doi:10.1038/s41467-023-36276-8)
Supplement: Supplementary file 1 — Supplementary Information [file 41467_2023_36276_MOESM1_ESM.pdf]

Supporting Information

Binuclear Cu complex Catalysis enabling Li–CO_2 battery with a high discharge voltage above 3.0 V

Xinyi Sun[1†], Xiaowei Mu[1†], Wei Zheng[2], Lei Wang[1], Sixie Yang[1], Chuanchao Sheng[1], Hui Pan[1], Wei Li[1], Chenghui Li[2], Ping He[1*] and Haoshen Zhou[1*]

[1]Center of Energy Storage Materials & Technology, College of Engineering and Applied Sciences, Jiangsu Key Laboratory of Artificial Functional Materials, National Laboratory of Solid State Microstructures and Collaborative Innovation Center of Advanced Microstructures, Nanjing University, Nanjing 210093, P. R. China.

[2]State Key Laboratory of Coordination Chemistry, School of Chemistry and Chemical Engineering, Nanjing National Laboratory of Microstructures, Collaborative Innovation Center of Advanced Microstructures, Nanjing University, Nanjing 210093, P. R. China.

[*]e-mail: pinghe@nju.edu.cn; hszhou@nju.edu.cn

[†]These authors contributed equally to this work.

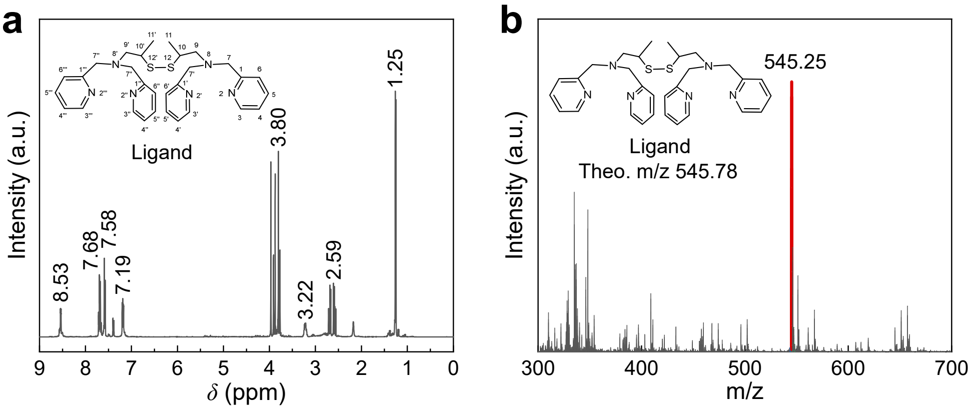

Supplementary Fig. 1 Characterizations of the ligand of Cu(I) RM. a, ^{1}H NMR spectrum and **b**, ESI-MS spectrum of the ligand of Cu(I) RM, whose structural formula is displayed in the insets of **a** and **b**.

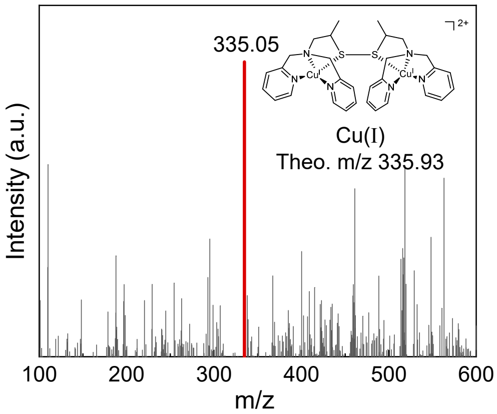

Supplementary Fig. 2 ESI-MS characterization of Cu(I) RM. ESI-MS spectrum of Cu(I) RM ($[Cu_2C_{30}H_{36}N_6S_2]^{2+}$), whose structural formula is depicted in the inset.

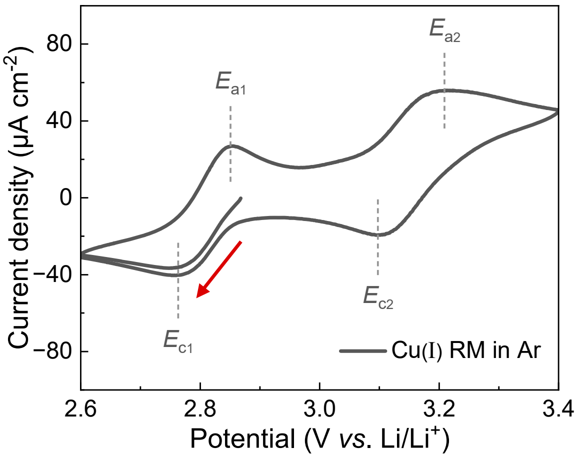

Supplementary Fig. 3 CV curve of Cu(I) RM. CV curve containing the first scanning cycle for the cell with 0.5 mM Cu(I) RM in 0.1 M $LiClO_4$/MeCN under Ar. Scan rate is 10 mV s^{-1}.

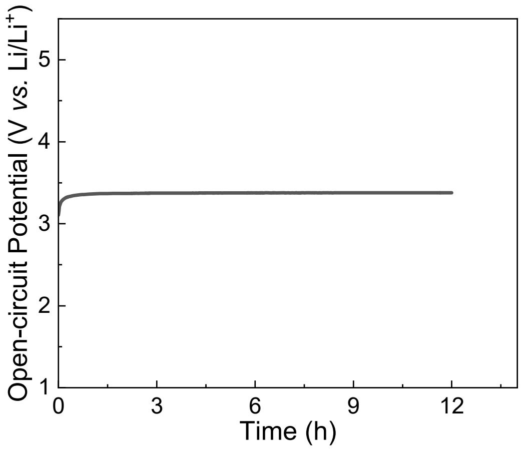

Supplementary Fig. 4 Open-circuit potential of Li–CO_2 battery containing Cu(I) RM. Open-circuit potential of the Li–CO_2 battery with Cu(I) RM-based electrolyte. Upon CO_2 pumping, the OCV experiences a rapid rising from 3.12 V to 3.38 V.

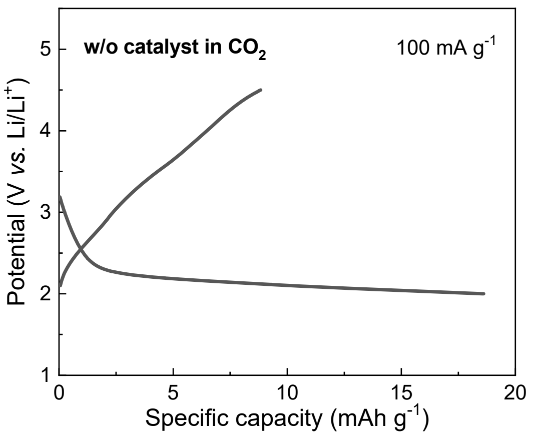

Supplementary Fig. 5 Electrochemical performance of conventional Li–CO_2 battery. Galvanostatic discharge-charge curve of the Li–CO_2 battery with a 0.1 M $LiClO_4$/MeCN electrolyte and a Super P carbon cathode at a current density of 100 mA g^{-1}. The Li–CO_2 battery exhibits an extremely small capacity of 19 mAh g^{-1}.

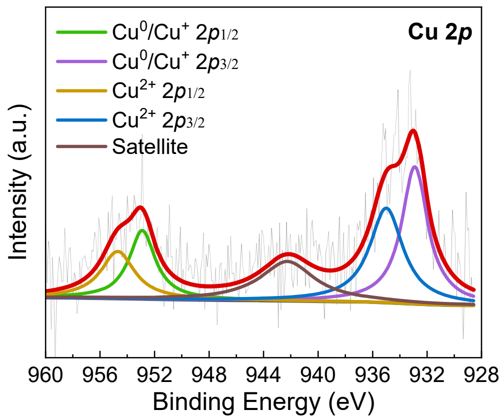

Supplementary Fig. 6 XPS characterization of discharged Super P carbon cathode. XPS spectrum of Cu 2*p* for discharged Super P carbon cathode in the Li–CO_2 battery with Cu(I) RM-based electrolyte, showing peaks assigned to Cu^0/Cu^+ and Cu^{2+}.

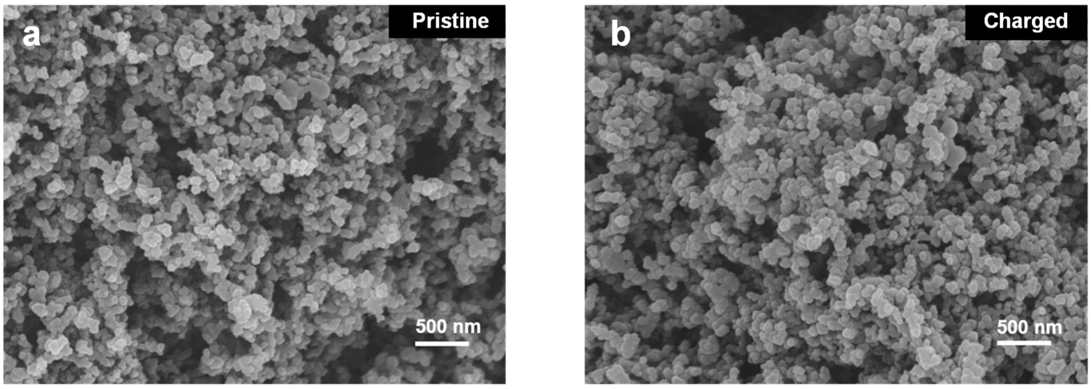

Supplementary Fig. 7 Morphology characterizations of pristine and charged Super P carbon cathodes. SEM images of **a**, pristine and **b**, recharged Super P carbon cathodes in Li–CO_2 batteries containing Cu(I) RM.

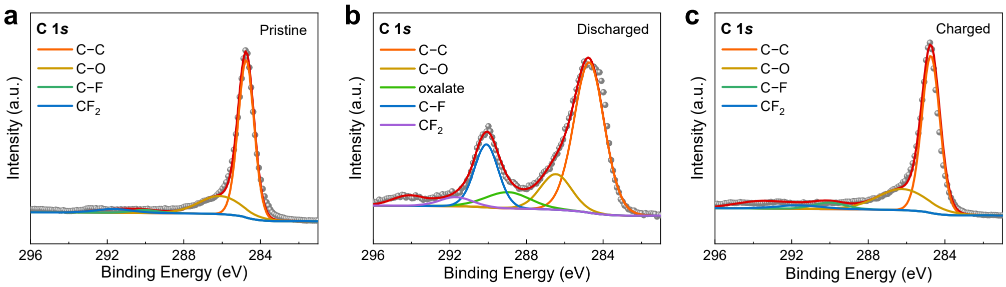

Supplementary Fig. 8 XPS characterizations of Super P carbon cathodes at different reaction stages. XPS C1*s* spectra of **a**, pristine, **b**, discharged, and **c**, recharged Super P carbon cathodes in Li–CO_2 batteries with Cu(I) RM-based electrolyte. They confirm the reversible production and decomposition of $Li_2C_2O_4$.

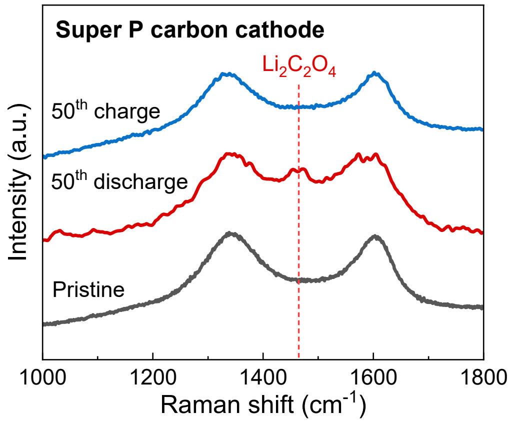

Supplementary Fig. 9 Raman characterizations of Super P carbon cathodes after 50 cycles. Raman spectra of Super P carbon cathodes at different reaction stages in Li–CO_2 batteries with Cu(I) RM after 50 cycles.

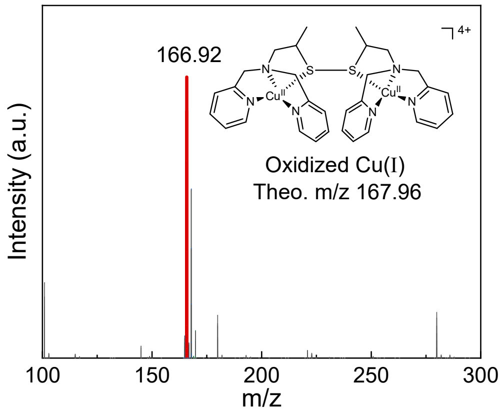

Supplementary Fig. 10 Electrolyte characterization after charge. ESI-MS spectrum of Cu(I) RM-based electrolyte after recharge, where the prominent peak can be assigned to the electrochemically oxidized Cu(I) RM ($[Cu_2C_{30}H_{36}N_6S_2]^{4+}$). The structural formula is depicted in the inset.

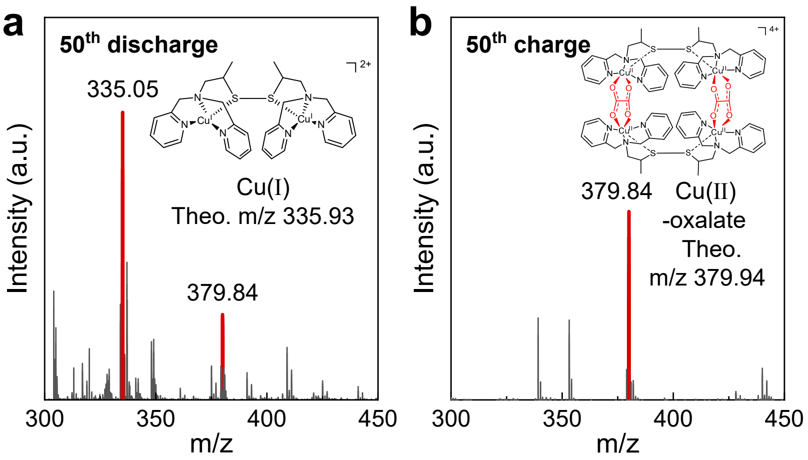

Supplementary Fig. 11 Electrolyte characterizations after 50 cycles. ESI-MS spectra of Cu(I) RM-based electrolytes in **a**, discharged and **b**, charged states after 50 cycles.

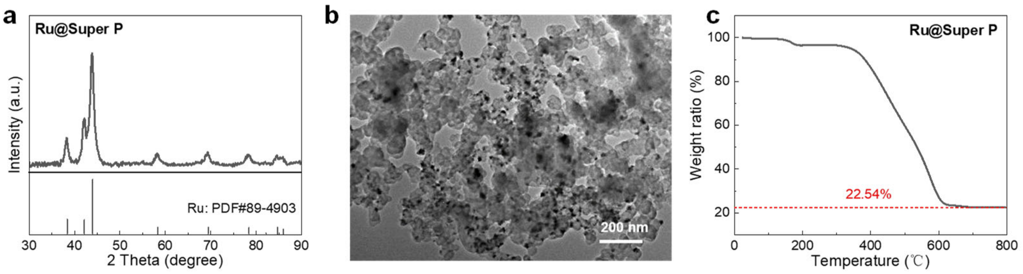

Supplementary Fig. 12 Characterizations of Ru@Super P material. a, XRD pattern, **b**, TEM image of the Ru@Super P powder. All peaks in the XRD pattern can be assigned to Ru nanoparticles (PDF#89-4903). The TEM image clearly exhibits that Ru nanoparticles are well-dispersed on Super P carbon. **c**, TG curve of the Ru@Super P material in O_2 gas. Temperature range is 25–800°C. Heating rate is 5°C min^{-1}.

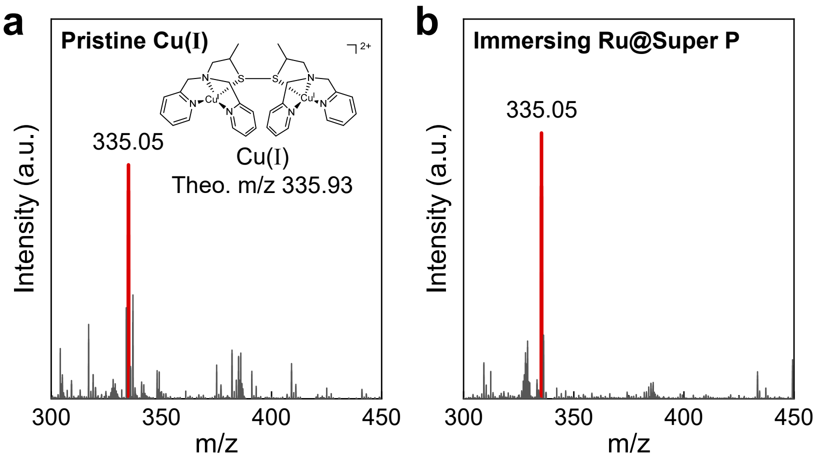

Supplementary Fig. 13 Electrolyte characterizations before and after immersing Ru@Super P cathode. ESI-MS spectra of Cu(I) RM-based electrolytes **a**, before and **b**, after immersing Ru@Super P cathode for 10 days.

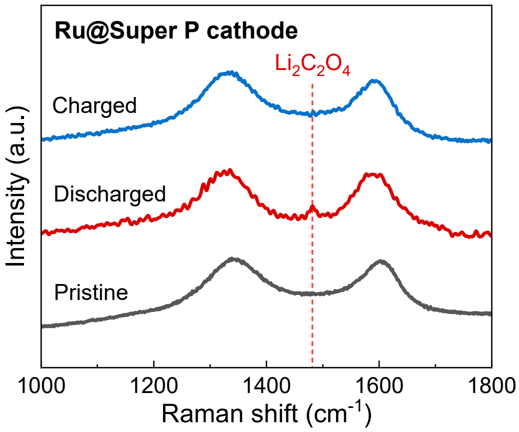

Supplementary Fig. 14 Raman characterizations of Ru@Super P cathodes at different reaction stages. Raman spectra of Ru@Super P cathodes at different reaction stages in Li–CO_2 batteries containing Cu(I) RM.

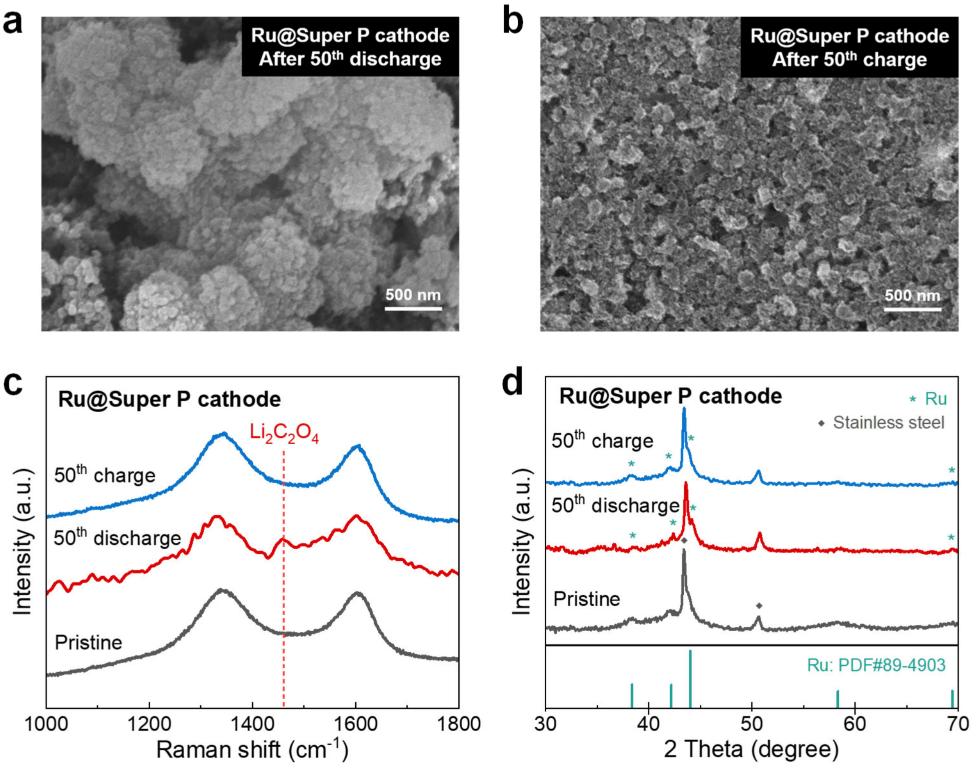

Supplementary Fig. 15 Characterizations of Ru@Super P cathodes after 50 cycles. SEM images of **a**, discharged and **b**, recharged Ru@Super P cathodes in Li–CO_2 batteries containing Cu(I) RM after 50 cycles. **c**, Raman and **d**, XRD patterns of Ru@Super P cathodes at different reaction stages in Cu(I) RM-based Li–CO_2 batteries after 50 cycles.

Supplementary methods

Charge-to-mass calculation of differential electrochemical mass spectrometry. The theoretical value of electrons (n_e) based on the charge capacity (Q_{th}) can be calculated as follows:

$$Q_{th} = It = 0.1\ \text{mA} \times 10\ \text{h} = 1\ \text{mAh} = 3.6\ \text{C}$$

$$n_e = \frac{Q_{th}}{Q_e \times N_A} = \frac{3.6\ \text{C}}{1.602 \times 10^{-19}\ \text{C} \times 6.02 \times 10^{23}\ \text{mol}^{-1}} = 3.73288 \times 10^{-5}\ \text{mol} = 37328.8\ \text{nmol}$$

Based on the practical CO_2 evolution quantity (n_{CO2}, 31280.9 nmol), the charge-to-mass ratio is

$$z/m = \frac{n_e}{n_{CO2}} = \frac{37328.8\ \text{nmol}}{31280.9\ \text{nmol}} = 1.19$$
